# The relationship between exacerbated diabetic peripheral neuropathy and metformin treatment in type 2 diabetes mellitus

**DOI:** 10.1038/s41598-021-81631-8

**Published:** 2021-01-21

**Authors:** Manal Mohammed Hashem, Ahmed Esmael, Abdelfattah Kasem Nassar, Mohammed El-Sherif

**Affiliations:** 1 Internal Medicine Department, Faculty of Medicine, Zagazig University, Zagazig, Egypt; 2 Neurology Department, Mansoura Faculty of Medicine, Mansoura University, Mansoura, 35516 Dakahlia Egypt; 3 Rheumatology Department, Physical Medicine and Rehabilitation Faculty of Medicine, Al-Azhar University, Cairo, Egypt

**Keywords:** Neuronal physiology, Peripheral neuropathies, Diabetes

## Abstract

Metformin-treated diabetics (MTD) showed a decrease in cobalamin, a rise in homocysteine, and methylmalonic acid, leading to accentuated diabetic peripheral neuropathy (DPN). This study aimed to determine whether or not metformin is a risk factor for DPN. We compared MTD to non-metformin-treated diabetics (NMTD) clinically using the Toronto Clinical Scoring System (TCSS), laboratory (methylmalonic acid, cobalamin, and homocysteine), and electrophysiological studies. Median homocysteine and methylmalonic acid levels in MTD vs. NMTD were 15.3 vs. 9.6 µmol/l; P < 0.001 and 0.25 vs. 0.13 µmol/l; P = 0.02, respectively with high statistical significance in MTD. There was a significantly lower plasma level of cobalamin in MTD than NMTD. Spearman’s correlation showed a significant negative correlation between cobalamin and increased dose of metformin and a significant positive correlation between TCSS and increased dose of metformin. Logistic regression analysis showed that MTD had significantly longer metformin use duration, higher metformin dose > 2 g, higher TCSS, lower plasma cobalamin, and significant higher homocysteine. Diabetics treated with metformin for prolonged duration and higher doses were associated with lower cobalamin and more severe DPN.

## Introduction

Diabetes mellitus (DM) is a chronic metabolic disease associated with hyperglycemia owing to impaired insulin secretion, insulin activity, or both^1^. High blood glucose levels can cause damage to all parts of the body including the cardiovascular system, eyes, kidneys, and nervous system^2^. American Diabetes Association 2019 recommended Metformin for the treatment of type 2 DM and should be continued as long as it is tolerated^3^. Nareddy et al. reported that metformin can cause cobalamin deficiency^4^. Cobalamin is associated with various metabolic functions, including red cell generation and intact nervous system, and so its dysfunction can cause anemia and neurological impairment^5,6^. Homocysteine is a sulfur-containing amino acid formed during the metabolism of methionine^7^. It is associated with cardiac and vascular illness produced in different ways like the increase of blood coagulation, oxidative stress, endothelial impairment, and cardiomyocyte impairment^8–10^. Bansal et al. concluded that atherosclerosis of blood vessels is the cause of most of DM related complications. Serum homocysteine can be utilized independently as an indicator of cardiovascular risk events in type 2 diabetes^11^. Subclinical cobalamin dysfunction is sometimes presented with ordinary blood levels^12^. Metformin is known to diminish serum cobalamin level with raised methylmalonic acid and homocysteine levels in patients with type 2 DM^13–16^. Diabetic peripheral neuropathy (DPN), is the most widely recognized complication of DM^17^. Liu et al. stated that the duration of diabetes, age, hemoglobin A1c, and diabetic retinopathy are associated with significantly increased risks of DPN among diabetic patients^18^. It affects approximately half of patients with diabetes^19^. The pathogenesis of DPN includes oxidative and inflammatory stress besides metabolic dysfunction causing neuronal injury and damage^20,21^. Electrophysiological nerve conduction studies (NCS) can confirm that the diagnosis of DPN and the sural nerve is the most applicable to correlating with the severity of DPN^22^. However, NCS does not generally distinguish early changes of neuronal damage and some DPN patients give negative results in the clinical assessment^23^. Our study intends to distinguish the serum level of cobalamin, methylmalonic acid and homocysteine changes in Metformin-treated diabetics (MTD) and to evaluate the connection between the severity of DPN and the long-term utilization of metformin.

## Methods

### Participants and inclusion criteria

150 adult patients with Type 2 DM (T2DM) were included according to Standards of Medical Care in Diabetes Guidelines^24^. We prospectively identified patients diagnosed with T2DM who were treated with metformin for more than 6 months. Also, we select non-metformin treated age and sex matched with metformin treated group with the same number of cases. The study design was a case–control, prospective, analytical, observational study. Cases were T2DM patients on metformin (75 participants). Controls were T2DM patients not taking metformin (75 participants).

The present prospective study was conducted during the period from the first of May 2017 to the end of September 2018 in the neurology outpatient clinic, Mansoura University Hospital, Egypt. The included patients were type two diabetics on oral hypoglycemic therapy with clinical proof for DPN. Patients were classified into 2 groups; Metformin treated (group I): included 75 patients administered metformin for the previous 6 months or more and non-metformin treated (group II): included 75 patients who weren’t administered metformin for the previous 6 months (but administered other oral hypoglycemic drugs). The duration of DPN was determined by the history of onset of symptoms or the previous electrophysiological studies done.

### Sample size determination

The formula for estimation of sample size is:

$${\text{n}} = \left( {{\text{Za2p }}\left( {{\text{1}}{-}{\text{p}}} \right)/{\text{e2}}} \right),$$

where **n** is the needed sample size, **Z** is the critical value at 95% confidence level (1.96), **p** is the prevalence of vitamin B12 deficiency in cases with type 2 DM on metformin (9.7%) obtained from the study of de Groot et al.^25^. **e** is the error margin that the researcher was willing to accept, and in this condition, was equal to 0.05. Note that (1–p) = **q**, which was the proportion of the sample population not covered by the study.

Substituting the values, we get n = 1.96 × 1.96 × 0.097 (1–0.097)/0.05 × 0.05 = 135 (with 10% attrition) 135 + 13.5 = 148.5. This was rounded up to 150 patients.

### Exclusion criteria

The patients excluded from this study possessed the following characteristics: type 1 diabetes mellitus, impaired glucose tolerance, peripheral neuropathy because of different causes other than diabetes or cobalamin insufficiency and patient cease treatment by metformin or administrated metformin for less than 6 months.

### Clinical assessment

Complete history taking for symptoms of DPN, family history of DPN, medical history, duration of DM, and current medications for DM or other systemic diseases was taken. Also, complete general and neurological examinations were done. The clinical assessment of the severity of DPN was done by the Toronto Clinical Scoring System (TCSS) which comprises of three sections: symptom scores, reflex scores, and sensory test scores. The greatest score is 19 points: 0–5 if there is no diabetic peripheral neuropathy, 6–8 if there is mild diabetic peripheral neuropathy, 9–11 if there is moderate diabetic peripheral neuropathy, 12–19 if there is severe diabetic peripheral neuropathy^24,26^.

### Electrophysiological studies and laboratory investigations

Regarding the electrophysiological studies, a nerve conduction study was carried out for all patients by utilizing Xilec-Xcalibur from Natus neurology, USA, 2010. The selected nerves, according to clinical symptoms and findings were examined for motor and sensory conduction studies, the distal latency, the amplitude of action potential, motor and sensory conduction velocities were measured^25,27^. The laboratory investigations include complete blood count, electrolytes, renal and hepatic functions, thyroid-stimulating hormone, rheumatoid factor, serum folate, Hemoglobin A1c, and serum cobalamin level according to the classic method of radioimmunoassay. High-performance liquid chromatography used to measure the homocysteine levels and mass spectrometry used to measure methylmalonic acid levels for all patients.

### Ethical approval

This study was approved by the Institutional Review Board (IRB) of the Mansoura Faculty of Medicine (proposal code R.19.10.640) and all participants gave informed consent to take part in the study. The research adhered to the tenets of the declaration of Helsinki (1964).

### Statistical analysis

The Statistical Package for Social Sciences (SPSS) programming, version 21.0 was utilized for information registration, approval, and analysis. Frequency, tables, and diagrams were produced for the categorical variables. Tests of significance were created for various variables. The Chi-square test was utilized to test categorical variables, and independent t-tests were utilized to test the importance of the results of the two groups. The Mann–Whitney U test was utilized to contrast and to investigate the means of the independent variables. The correlation coefficient and Chi-squared tests were utilized to gauge the relation between two quantitative and qualitative variables. The degree of statistical significance was characterized by an estimation of P-value < 0.05. Spearman’s correlation coefficient was done to analyze the relationship between continuous variables such as metformin dose and vitamin B12 levels and also between metformin dose and TCSS scores. Logistic regression analysis was carried out to identify independent risk factors for diabetic PN in metformin-treated patients.

## Results

### Patients demographic and clinical characteristics

This study was carried out on 150 patients with type 2 diabetes mellitus. Group I included 75 patients administered metformin for the previous 6 months or more. Group II included 75 patients who weren’t administered metformin for the previous 6 months (but administered other oral hypoglycemic drugs). The two groups were similar in age, sex. Also, there was no important difference between both groups regarding differences in the disease severity, the duration of diabetes, and duration of diabetic PN (P = 0.9 and 0.82 respectively (Table 1). MTD had a significantly higher moderate to severe DPN and higher total scores of TCSS (10 ± 7.5 vs. 5 ± 9.5, P < 0.001) indicated that metformin users had a more severe DPN (Table 1).

**Table 1:** Patients’ demographic and clinical characteristics, divided according to metformin use (N = 150).

Variables	MT N (75)	NMT N (75)	P-value
Age (years)	56.9 ± 12.3	54.3 ± 10.9	0.91
Male sex (%)	41 (54.7)	39 (52)	0.7434
Duration of DM (years)	5.9 ± 4.3	3.9 ± 4.8	0.995
SBP (mmHg)	129.9 ± 18.72)	127.82 ± 19.2	0.59
DBP (mmHg)	76.81 ± 8.7	75.0 ± 10.1	0.74
BMI (kg/m^2^)	31.9 ± 8.43	32.73 ± 7.92	0.88
Duration of DPN (years)	3.2 ± 2.1	2.9 ± 1.8	0.82
Diagnosed neuropathy N (%)	38 (50.7%)	20 (26.7%)	0.002
**TCSS**			
Total TCSS score	10 ± 7.5	5 ± 9.5	< 0.001
No DPN	37 (49.3%)	55 (73.3%)	< 0.001
Mild	12 (16%)	10 (13.3%)	
Moderate	19 (25.3)	7 (9.4%)	
Severe	7 (9.4%)	3 (4%)	
**Duration of metformin intake**			
< 1 year	15 (20) %	N/A	N/A
1–4 years	29 (38.7%)	N/A	
> 4 years	31 (41.3%)	N/A	
**Metformin dose**			
< 1000 (mg)	15 (20%)	N/A	N/A
1000–2000 (mg)	50 (66.7%)	N/A	
> 2000 (mg)	10 (13.3%)	N/A	

MT: metformin-treated, NMT: non-metformin-treated, SBP: systolic blood pressure, DBP: diastolic blood pressure, BMI: Body Mass Index, TCSS: Toronto clinical scoring system, DPN: diabetic peripheral neuropathy, N/A: not applicable.

### Laboratory findings for metformin-treated and non-metformin-treated groups

Table 2 showed a lower median serum cobalamin in the metformin-treated diabetics with high statistical significance (222 vs. 471 pmol/l; P < 0.001). There were also significant differences between the two groups in median serum homocysteine and methylmalonic acid levels, and MTD showed higher levels with P < 0.05. HbA1c was higher in metformin-treated diabetics without statistical significance (P = 0.09).

**Table 2:** Comparison of laboratory features for metformin-treated and non-metformin-treated groups.

Laboratory features	MT N (75)	NMT N (75)	P-value
Serum Cbl (pmol/L) median (range)	222 (337)	471 (799)	< 0.001
Cbl deficiency (< 210 pmol/L) N (%)	25 (33%)	3 (4%)	< 0.001
Fasting serum Hcy (µmol/L) median (range)	15.3 (19.6)	9.6 (16.3)	< 0.05
Fasting serum MMA (µmol/L) median (range)	0.25 (0.61)	0.13 (0.21)	< 0.05
HbA1c	7.6 ± 1.1	6.7 ± 1.0	0.09

MT: metformin-treated, NMT: non-metformin-treated, Cbl: cobalamin, Hcy: homocysteine, MMA: methylmalonic acid, N: number, HbA1c: hemoglobin A1c.

### Electrophysiological study of both groups

Median conduction velocity for superficial peroneal and sural nerves in MTD showed significant slower conductivity with P < 0.05. Additionally, median sensory nerve action potentials (SNAP) studies for sural and superficial peroneal nerves in the MTD showed a significantly lower level with P < 0.05 (Table 3).

**Table 3:** Comparison of nerve conduction study features for metformin-treated and non-metformin-treated groups.

Electrophysiological findings	MT N (75)	NMT N (75)	P-value
Superficial peroneal nerve SNAP (amplitude µv)	3.2 ± 5.7	5.9 ± 6.9	< 0.05
Superficial peroneal nerve sensory conduction velocity (m/s)	31.1 ± 7.1	35.0 ± 8.5	< 0.05
Sural nerve SNAP (amplitude µv)	3.3 ± 6.1	6.1 ± 7.5	< 0.05
Sural nerve sensory conduction velocity (m/s)	32.9 ± 9.1	36.1 ± 7.7	< 0.05

SNAP: sensory nerve action potential, MT: metformin-treated, NMT: non-metformin-treated, N: number.

### Correlation of metformin dose to vitamin B12 and Cbl plasma levels

**Figure 1:**
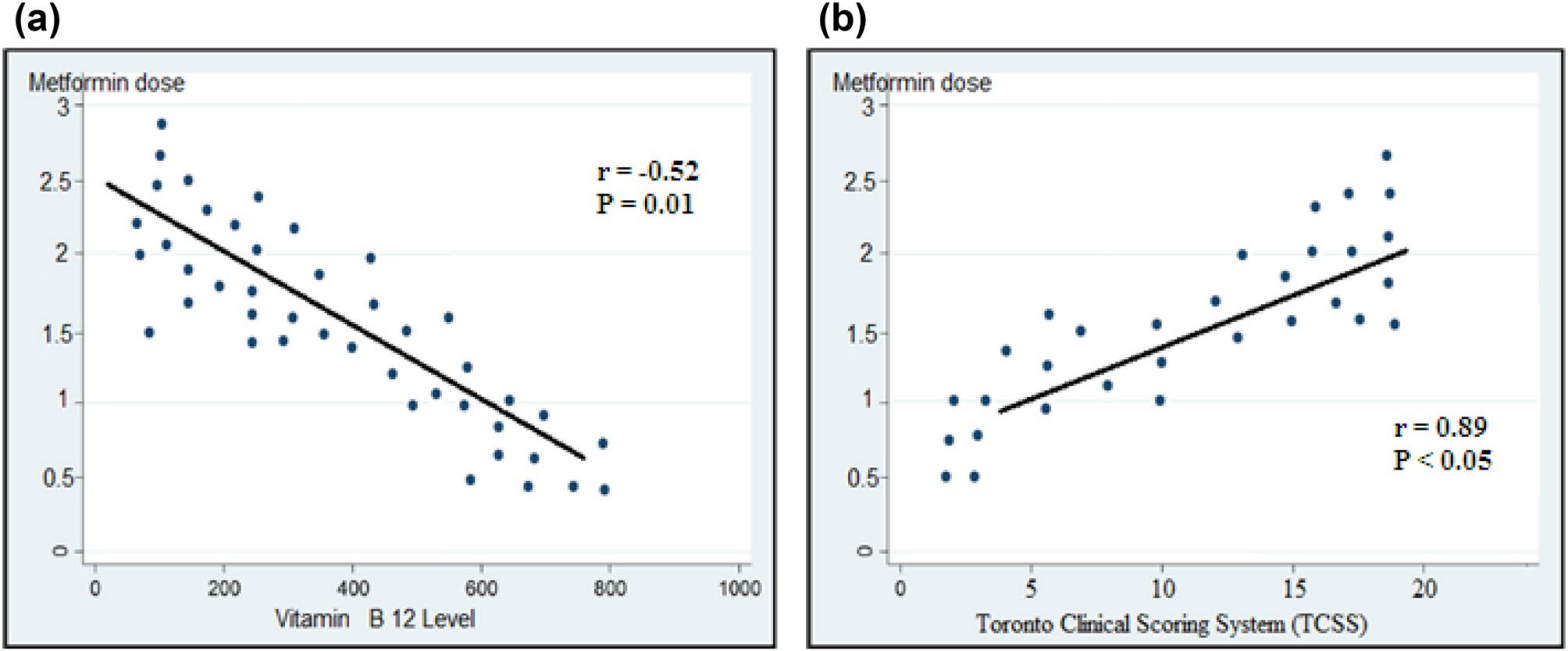
(**A**) Correlation of vitamin B12 and metformin dose. (**B**) Correlation of Toronto Clinical Scoring System and metformin dose.

Spearman’s correlation coefficient was used to examine the bivariate relationship between the two continuous variables of metformin dose and vitamin B12 levels and metformin dose and TCSS. Figure 1A showed a significant negative correlation between cobalamin plasma levels and higher doses of metformin (r =  − 0.522 and P = 0.01). Figure 1B showed a significant positive correlation between TCSS and increased dose of metformin (r = 0.891 and P < 0.05).

### Correlation of the severity of DPN with cobalamin, homocysteine, and methylmalonic acid (Fig. 2)

**Figure 2:**
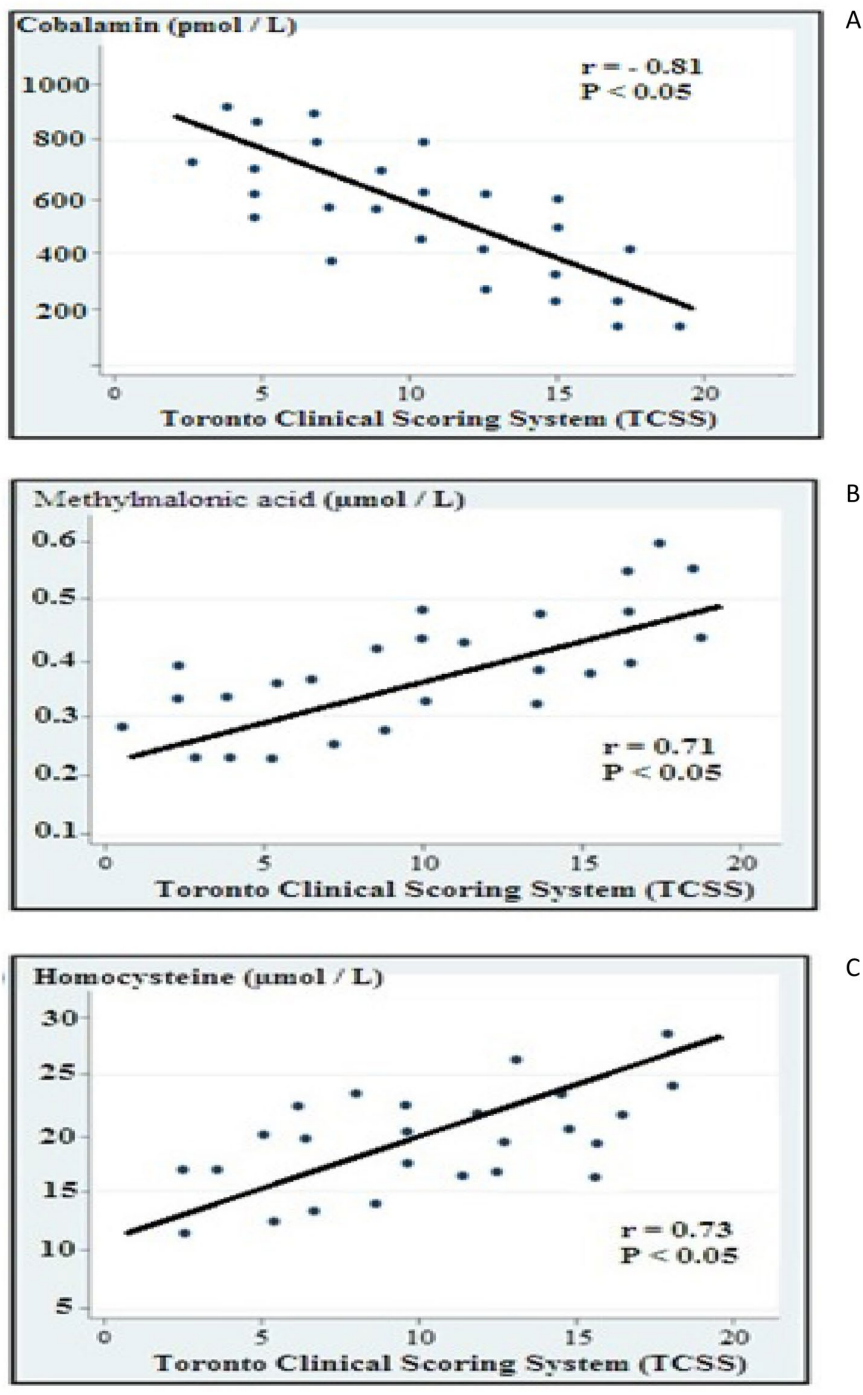
Correlation of the severity of DPN with (**A**) cobalamine, (**B**) methymalonic acid, and (**C**) homocysteine.

There was a significant inverse relationship with the severity of DPN and cobalamine level (r = − 0.81 and P < 0.05), while the severity of DPN was directly related to higher levels of both methylmalonic acid, and homocysteine (r = 0.71 and P < 0.05; and r = 0.73 and P < 0.05 respectively).

### Independent predictors of diabetic peripheral neuropathy in metformin-treated diabetics

Finally, multivariate logistic regression analysis of independent predictors of DPN in MTD showed that longer duration of DM and treatment by metformin were significantly associated with more incidence of DPN (OR = 1.2% CI 1.1–1.3 and P < 0.05 and OR = 1.8 CI 1.1–2.5, P < 0.05 respectively). Also, larger doses and longer duration of therapy by metformin were independent predictors of diabetic peripheral neuropathy (OR = 6.43% CI 1.39–13.94 and P < 0.01 and OR = 5.89 CI 1.34–15.92, P = 0.01 respectively). Moreover the Logistic regression analysis showed that metformin-treated patients had significantly lower plasma cobalamin (OR = 3.67% CI 0.91 to 8.96, P < 0.05) and significantly higher homocysteine (OR = 7. 45% CI 2.16 to 15.77, P < (Table 4).

**Table 4:** Multivariate logistic regression analysis of independent predictors of diabetic peripheral neuropathy in metformin-treated diabetics.

	Odds ratio (95% CI)	P-value
Duration of diabetes	1.2 (1.1–1.3)	< 0.05
Drug treatment—metformin	1.8 (1.1–2.5)	< 0.05
**Metformin duration (years)**		
< 1 year	1.23 (0.82–2.58)	0.09
1–4 years	2.47 (0.87–9.64)	< 0.05
> 4 years	5.98 (1.34–15.92)	0.01
**Daily dose of metformin (g)**		
< 1000	1.69 (0.71–2.11)	0.08
1000–2000	3.91 (0.95–9.61)	< 0.05
> 2000	6.43 (1.93–13.94)	< 0.01
Homocysteine (μmol/L)	7. 45 (2.16–15.77)	< 0.01
Serum Cbl (μmol/L)	3.67 (0.91–8.96)	< 0.05
**Toronto clinical scoring system**		
No DPN	Ref	
Mild DPN	1.23 (0.82–2.58)	0.09
Moderate DPN	3.73 (0.86–7.98)	0.05
Severe DPN	6.21 (1.85–11.79)	< 0.05

DPN: diabetic peripheral neuropathy, Cbl: cobalamin, DPN: diabetic peripheral neuropathy.

## Discussion

Diabetic patients have different degrees of nervous system damage ranging from mild to severe forms, the most widely recognized being peripheral neuropathy which affects 60% to 70% of cases^28^. The aim of this study was to assess whether metformin is a risk factor for the development of DPN by evaluating the impact of metformin on the serum level of cobalamin and homocysteine in type 2 DM. Most of the past clinical studies demonstrated that metformin has some impact on plasma cobalamin and homocysteine and there are some case reports indicating deterioration of neuropathy with metformin^29–32^. Some recent researches have investigated the relation of metformin with the level of cobalamin and neuropathy with conflicting results^29,33,34^.

The present single-center prospective study during a one and half year duration was carried out on 150 patients (80 males and 70 females) with DPN, during their attendance to the neurology outpatient clinic. The current study stated that the MTD had significant higher DPN (50.7%), total TCSS scores (10 ± 7.5), serum levels of homocysteine (15.3 µmol/l) and methylmalonic acid (0.25 µmol/l), lower cobalamin (222 pmol/l), median conduction velocity and SNAP for superficial peroneal, and sural nerves. Also, there was a significant negative correlation between cobalamin and metformin. On the other hand, there was a significant positive correlation between TCSS and metformin.

The significant predictors' explanatory parameters associated with DPN occurrence in MTD were larger dose and longer duration of metformin usage, low cobalamin level, and high homocysteine level. Our results were in harmony with Khan et al. who concluded that patients with type 2 DM with prolonged duration of treatment with metformin were accompanied by a higher occurrence of vitamin B12 inadequacy^35^. So, clinicians should screen diabetic patients who are metformin users for any B12 deficiency accordingly. Our study exhibited a higher median serum level of Hcy and MMA. These variations from the normal values were highly related to metformin users. Our findings were as per that of Ahmed and Chapman et al., demonstrating that long-term metformin users compared to the non- metformin users had lower serum Cbl, higher MMA and Hcy^36,37^.

Our results demonstrate that metformin treatment conveys a potential hazard for the occurrence of cobalamin lack. However, this would most likely be little if cobalamin status is monitored consistently. Cobalamin inadequacy is increased among patients with type 2 diabetes mellitus. There is a requirement for a more precise differential conclusion to recognize diabetic neuropathy and metformin-induced neuropathy. We also found that patients with type 2 DM, DPN, and more than 6 months of treatment by metformin demonstrated higher scores on the TCSS, indicating more clinically severe DPN when contrasted with comparative to non-metformin users. These findings were frequently connected with cumulative metformin utilization with a positive correlation between DPN and cumulative metformin dose. These results were similar to that of Holay et al. who found the incidence of neuropathy by TCSS and NCV test was significantly higher in metformin users with a positive correlation with cumulative dose and duration of metformin and a negative correlation with serum vitamin B12 levels^38^. Similar to our results, Singh et al. discovered a smaller yet significant difference in neuropathy scores in between metformin and non-metformin users^39^.

Electrophysiologically, this study exhibited that the metformin users had more severe DPN. Measurements of the mean conduction velocity and mean amplitude of SNAP of the examined nerves were altogether significantly lower in the metformin users’ group. These results are not quite the same as Wile and Toth who found an insignificant lower mean conduction velocity and mean amplitude of SNAP in the metformin users’ group^40^. This may be explained by the difference in demographic characteristics and longer duration of the disease in our study. The nerves of lower limbs, especially sural and superficial peroneal nerves were more sensitive to diabetic effects. This may be also supported by the study of Shibata et al. and Agarwal et al. who found that the sural nerve conduction study had the most reliable test that correlates well with the severity of diabetic PN^41,42^.

In our study the severity of DPN was inversely correlated with the cobalamine level and directly correlated with higher levels of both methylmalonic acid, and homocysteine. Similarly, Wile and Toth found that the clinically worsened DPN was associated with lower cobalamine level and higher levels of both methylmalonic acid, and homocysteine^40^. It has been shown that elevated levels of total homocysteine significantly correlated with DPN, independent of other risk factors^43,44^. The serum MMA positively correlated with the severity of neuropathic pain and this can be used as a useful marker in assessment of peripheral neuropathy^45^.

The discoveries in this study recommended that a cumulative dose of metformin is accompanied with a decrease in levels of cobalamin and deterioration of DPN. However, metformin is the main therapy in type 2 DM and has appeared to have different valuable impacts on diabetes patients accomplished by changing advanced glycosylation end products and neurodegenerative processes^46,47^. Hence, we advise that serum cobalamin levels should be regularly checked on Type 2 DM patients on long term metformin therapy and at higher risk of DPN. Finally, logistic regression analysis for risk factors of diabetic PN in metformin-treated patients discovered that longer metformin use duration (5.98 vs. 4 years, P = 0.01), higher metformin dose (6.43 vs.  > 2000 g, P = 0.01), lower plasma level of cobalamin (OR = 3.67% CI 0.91 to 8.96, P < 0.05) and duration of diabetes (OR in = 1.2% CI 1.1 to 1.3, P < 0.05) were the most important independent factors of diabetic PN. This was similar to the comment of Alharbi, et al. that the duration and dose of metformin usage were related to the decline in levels of cobalamin. Moreover, as cobalamin has a crucial role in the homocysteine metabolism, a decrease in cobalamin would elevate the plasma level of homocysteine^31^. However Ahmed et al. published a cross-sectional study where vitamin B12 has measured 121 type 2 diabetics and the association with DPN was evaluated. However, there was no association between vitamin B12 deficiency and diabetic PN, and Metformin dose did not confer an increased risk on diabetic PN presence^48^. Similar results were reported by other authors, with controversial results, but without strong evidence that vitamin B12 deficiency influences the presence or severity of peripheral neuropathy^29,49^. But our results showed a positive correlation between metformin-treated patients and severity of DPN as showed by a linear correlation with TCSS (r = 0.891 and P < 0.05). Yang et al. stated that Metformin uses led to decreased cobalamin levels in diabetic patients. More research is needed to investigate the correlation between metformin use and neuropathy in diabetics and annual cobalamin measurement in MTD is recommended^50^.

### Limitations and directions of future work

Finally, the present study had some limitations. The first limitation was the relatively small number of patients due to the limitation of resources and financial issues. Glycemic control not being assessed is also a limitation of this study.

Also, this study, cannot evaluate long-term effects of risk factors concerning the development of DPN. Further studies should be conducted on a larger number of patients for a longer duration and to follow up patients with DPN for the improvement of manifestations especially after stoppage of metformin.

## Conclusion

Patients with type 2 DM who were treated with metformin for prolonged duration and higher doses were associated with lower plasma cobalamin and more severe DPN. Patients on metformin treatment must monitor regularly for plasma levels of cobalamin, methylmalonic acid, and homocysteine.

## Recommendation

- Routine screening of type 2 diabetic patients on metformin for vitamin B12 inadequacy is highly recommended due to its high prevalence and the significant clinical impacts that may result in DPN.
- Besides, we suggest, beginning treating patients with B12 once a borderline or low level is recognized.
